# Mitochondria in the Center of Human Eosinophil Apoptosis and Survival

**DOI:** 10.3390/ijms15033952

**Published:** 2014-03-05

**Authors:** Pinja Ilmarinen, Eeva Moilanen, Hannu Kankaanranta

**Affiliations:** 1 The Immunopharmacology Research Group, University of Tampere School of Medicine and Tampere University Hospital, Tampere FI-33014, Finland; E-Mails: eeva.moilanen@uta.fi (E.M.); hannu.kankaanranta@epshp.fi (H.K.); 2 Department of Respiratory Medicine, Seinäjoki Central Hospital, Seinäjoki, Finland and University of Tampere, Tampere FI-60220, Finland

**Keywords:** asthma, eosinophil, apoptosis, survival, mitochondrial permeability transition, reactive oxygen species, mitochondria, glucocorticoids, nitric oxide, mitogen-activated protein kinase

## Abstract

Eosinophils are abundantly present in most phenotypes of asthma and they contribute to the maintenance and exacerbations of the disease. Regulators of eosinophil longevity play critical roles in determining whether eosinophils accumulate into the airways of asthmatics. Several cytokines enhance eosinophil survival promoting eosinophilic airway inflammation while for example glucocorticoids, the most important anti-inflammatory drugs used to treat asthma, promote the intrinsic pathway of eosinophil apoptosis and by this mechanism contribute to the resolution of eosinophilic airway inflammation. Mitochondria seem to play central roles in both intrinsic mitochondrion-centered and extrinsic receptor-mediated pathways of apoptosis in eosinophils. Mitochondria may also be important for survival signalling. In addition to glucocorticoids, another important agent that regulates human eosinophil longevity via mitochondrial route is nitric oxide, which is present in increased amounts in the airways of asthmatics. Nitric oxide seems to be able to trigger both survival and apoptosis in eosinophils. This review discusses the current evidence of the mechanisms of induced eosinophil apoptosis and survival focusing on the role of mitochondria and clinically relevant stimulants, such as glucocorticoids and nitric oxide.

## 1. Introduction

Eosinophils are cells of the innate immune system involved in the pathogenesis of allergic, gastrointestinal and hypereosinophilic disorders, in anti-parasitic defence and in tumor immunity [1–5]. Eosinophils account only for approximately 3% of blood leukocytes in healthy individuals but their number is elevated in subjects with eosinophilic conditions such as allergic asthma. However, shortages of cells often hamper studies on eosinophils. In asthmatic airways, eosinophils are driven into an activated state e.g., by pro-inflammatory cytokines such as IL-5. When activated, they release toxic and pro-inflammatory mediators able to induce bronchoconstriction, mucus hypersecretion, and damage to airway epithelium as well as contribute to T helper 2 cell polarization and airway thickening [6–8]. Recent data from clinical studies with anti-IL-5 antibody and eosinophil-deficient mice suggest that eosinophils are important for asthma exacerbations and airway remodelling. Anti-IL-5 treatment led to decreased exacerbation rate in patients with severe eosinophilic asthma and these patients were able to reduce their glucocorticoid dose in response to anti-IL-5 treatment [9,10]. Thereby, eosinophils are important for maintenance and exacerbations of asthma [11].

In healthy individuals eosinophils are short-living cells. In *in vitro* conditions, blood eosinophils undergo spontaneous apoptosis in a few days but in a physiological situation they tend to migrate and accumulate into liver and spleen, where they are likely to live longer than a few days [12–15]. Apoptosis of eosinophils can be delayed or accelerated by various agents [14,15]. Eosinophil longevity may be enhanced up to 1–2 weeks by pro-inflammatory cytokines such as IL-5, IL-3 and GM-CSF present in inflamed airways [16]. Indeed, blood and tissue eosinophils from patients with asthma have been shown to live longer when compared to eosinophils from healthy individuals [17,18]. Eosinophil removal from the airways is beneficial to reduce eosinophilic inflammation and alleviate symptoms of asthma [19]. Apoptosis is a non-inflammatory way of cell death comprising a beneficial means for cell removal. Membrane integrity is retained throughout the process and the harmful cell content maintained inside the cell. The immunological silence of apoptosis is ensured by formation of smaller apoptotic bodies that are rapidly ingested by phagocytes. Apoptosis may be executed via two different main routes, extrinsic (receptor-mediated) or intrinsic (mitochondrion-centered) pathway [20]. Extrinsic pathway is activated e.g., by ligation of the death receptor Fas/CD95. This leads to formation of a multiprotein complex called death-inducing signalling complex (DISC) that regulates activation of initiator caspase-8. Initiator caspase-8 may either directly activate effector caspases that execute apoptosis or cleave BH3-interacting-domain death agonist (Bid) resulting in activation of an additional mitochondrial loop. Intrinsic pathway can be initiated by several intracellular stress conditions such as DNA damage, oxidative stress and cytosolic Ca^2+^ overload. Members of the Bcl-2 family are critical in monitoring intracellular damage and aim to mediate activation of pore-forming Bax and the following mitochondrial membrane permeabilization (MMP), a central event in apoptosis [20,21]. Mitochondrial membrane permeabilization can also be mediated via mitochondrial permeability transition (mPT) [21,22]. MMP results in loss of mitochondrial membrane potential (ΔΨ_m_), halted mitochondrial ATP synthesis and release of pro-apoptotic proteins such as cytochrome c to the cytosol. Cytochrome c stimulates formation of the apoptosome, a platform that activates initiator caspase-9 [20,21]. Initiator caspase 9 activates effector caspases 3, 6 and 7 resulting in degradation of cellular components and apoptosis.

Eosinophil apoptosis can be accelerated by physiological factors such as Fas activation [23]. Fas ligand is a significant pro-apoptotic agent for eosinophils *in vivo* because its neutralization enhanced airway eosinophilia in a mouse model of allergic asthma [24]. NO is produced in high amounts in the lungs of asthmatics and has been shown to regulate eosinophil apoptosis in a complex manner. NO has shown both anti- and pro-apoptotic effects on eosinophils [25–27] and both enhancing and reducing properties regarding lung eosinophilia [28–30]. Thereby, the net effect in response to NO can be different in different pathophysiological situations and is not known at the moment. Also many anti-asthmatic agents such as glucocorticoids, theophylline and cysteinyl leukotriene receptor antagonists enhance eosinophil apoptosis in the absence and presence of eosinophil survival-prolonging cytokines [31–36] and the pro-apoptotic effects of these drugs may contribute to their clinical efficacy [37–42]. Anti-inflammatory glucocorticoid medication is the corner stone in the treatment of asthma and understanding its actions is of critical importance. Glucocorticoids modulate longevity of many immune cell types and the sensitivity to glucocorticoid-induced cell death depends on the cell type. For example, CD4+ T cells but not CD8+ T cells are sensitive to glucocorticoid-induced apoptosis [43–45]. In contrast, glucocorticoids inhibited neutrophil apoptosis [31,46–48], even though not in an environment with severe hypoxia [49]. In eosinophils, glucocorticoids accelerate apoptosis [31–33].

Understanding the signalling related to eosinophil survival and apoptosis is extremely important for understanding the pathogenesis of eosinophilic inflammation and for the development of novel drugs to treat diseases associated with eosinophilia. Studies using primary human eosinophils are hampered by several issues. Low numbers of cells available for the studies restricts carrying out experiments with long-time series and different treatments. Additionally, the short lifespan of eosinophils excludes use of most modern molecular biology methods such as transfection and RNA interference. Because of the restrictions, knowledge of primary human eosinophil functions is based mainly on the use of pharmacological inhibitors and methods available for direct measurements of cellular functions and intracellular mediators.

In eosinophils, mitochondria play a central role in apoptosis and survival [50]. Mitochondrial events have been shown to be critical for spontaneous, glucocorticoid-, nitric oxide- and anti-Fas-induced apoptosis of eosinophils [51–54] and thereby mediate both extrinsic and intrinsic forms of eosinophil apoptosis. Mitochondria are forums where pro- and anti-apoptotic signals merge and the fate of the cell is determined. Because of the evident importance of this cell organ for eosinophil survival and death, this review concentrates on discussing the mechanisms of eosinophil apoptosis and survival focusing on the role of mitochondria and the clinically relevant pro-apoptotic stimulant glucocorticoid.

## 2. Mitochondria and Bcl-2 Family Members

In most cells, mitochondria function as “energy factories” producing ATP via function of the electron transport chain maintaining mitochondrial membrane potential (ΔΨ_m_). Eosinophils have been suggested to contain only a low number of mitochondria, 24–36 per cells [50] but this result needs to be confirmed by current techniques. It has also been reported that eosinophils maintain mitochondrial membrane potential rather by hydrolysis of ATP than via respiratory chain [50], even though evidence also exists of functional respiratory chains in eosinophils [55]. It is clear that eosinophil mitochondria are able to release cytochrome c from the intermembrane space and by this mechanism activate caspases and induce apoptosis [50].

### 2.1. Bcl-2 Members and Pore-Forming Activity of Bax and Bid

Members of the Bcl-2 family are critical in monitoring intracellular damage and the balance between anti-apoptotic and pro-apoptotic Bcl-2 members (ratio of pro-apoptotic Bax to anti-apoptotic Bcl-2) determines the susceptibility of cells to apoptosis. Short-living granulocytes have high ratios of Bax/Bcl-2 while the corresponding ratios in monocytes and lymphocytes are relatively low. This ratio determined the susceptibility of leukocytes to anti-Fas-induced apoptosis, granulocytes being the most susceptible and lymphocytes the least susceptible [56]. Eosinophils express high levels of pro-apoptotic Bid and Bax, which are proteins capable to oligomerize and form pores to the mitochondrial outer membrane [53,54,57,58]. Bid is processed during spontaneous apoptosis and in enhanced manner during glucocorticoid- and anti-Fas-induced apoptosis but it is the most critical mediator for Fas-induced apoptosis [53,54]. Also Bax was demonstrated to spontaneously translocate into mitochondria in untreated eosinophils and in an accelerated manner in response to glucocorticoid treatment [51,52]. Peptidyl-prolyl isomerase Pin1 may be the key regulator of Bax translocation. Bax translocation into mitochondria was prevented by GM-CSF, which activated ERK1/2 to phosphorylate the threonine residue of Bax. This phosphorylation enabled interaction of Pin1 with Bax preventing its mitochondrial targeting [58]. Pin1 was also shown to be a key regulator of apoptosis induced by anti-Fas via the Fas-associated death domain (FADD) in activated eosinophils. In the presence of survival-prolonging IL-5, Pin1 quenched phosphorylation of FADD at Ser^194^ and prevented apoptosis [59]. These mechanisms may be highly important in determining whether allergic inflammation is continued by IL-5/GM-CSF or diminished by Fas receptor signalling.

Anti-apoptotic Mcl-1 has been found in eosinophils and was degraded during spontaneous apoptosis and in an accelerated manner during apoptosis induced by glucocorticoids or by an inhibitor of cyclin-dependent kinase [57,60–63]. In HeLa cells, Mcl-1 was demonstrated to inhibit Bax downstream to its mitochondrial translocation. Mcl-1 prevented formation of Bax oligomers at mitochondria, required for pore formation, but this inhibition required no direct interaction [64]. Thereby, degradation of Mcl-1 in apoptotic eosinophils probably enables pore-forming activity of Bax and mitochondrial outer membrane permeabilization. The mechanism of Mcl-1 degradation accelerated by glucocorticoids remains unclear but may for example involve transcription of proteins involved in the degradation [63]. In neutrophils, glucocorticoids, in contrast to eosinophils, induced Mcl-1 expression, which may play an important role in the mechanism of prolongation of neutrophil survival [65]. Contradictions exist concerning the expression of anti-apoptotic Bcl-2 in eosinophils; expression seems to depend on the origin of the eosinophils [39,57,60,66].

### 2.2. Mitochondrial Permeability Transition

Mitochondrial permeability transition (mPT) is one mechanism for the mitochondrial membrane permeabilization. During mPT, permeability of the inner mitochondrial membrane is increased for solutes and molecules up to 1.5 kDa. A channel sensitive to Ca^2+^, oxidants and pro-apoptotic Bcl-2 family members is responsible for this phenomenon [21,22]. Mitochondrial permeability transition results in mitochondrial matrix swelling, most likely due to the influx of ions that are accompanied by water. The mitochondrial outer membrane is ruptured due to matrix swelling and apoptosis-inducing proteins are released to the cytosol [67]. The mPT channel is thought to be a multiprotein complex but its molecular structure is still unknown. Glucocorticoids and nitric oxide induced apoptosis in eosinophils that was mediated by mPT [68,69]. However, mPT had no critical role in mediating spontaneous or anti-Fas-induced apoptosis [23]. What defines the mechanism of mitochondrial membrane permeabilization in response to different pro-apoptotic stimulants in eosinophils remains unclear. Oxidants are generally important mediators of eosinophil apoptosis and known inducers of mPT but do not, however, always mediate mPT. It seems that ROS have several mechanisms to mediate apoptosis, one of which is stimulation of mPT. For example, Bid has been shown to engage a ROS-dependent but mPT-independent mechanism for mitochondrial membrane permeabilization and cytochrome c release [70].

Mitochondrial permeability transition may function in two different modes. In addition to the irreversible sustained opening of the mPT channel occurring during cell death, the channel may also fluctuate between open and closed states (flicker) [71,72]. In NO-treated eosinophils, early flickering mPT preceded permanent mPT and mPT-dependent eosinophil apoptosis [68]. The early flickering mPT was not necessary for apoptosis to proceed and may actually represent a cell survival mechanism [73,74]. It has been demonstrated that flickering mPT may act as a mechanism to release ROS or calcium [75–77] and by these mechanisms, flickering mPT may participate in cell signalling. For example, in NO-treated eosinophils flickering mPT mediated activation of JNK [68].

## 3. Reactive Oxygen Species (ROS) and Pro-Apoptotic Signalling Pathways

Reactive oxygen species such as superoxide O_2_^•−^ and hydrogen peroxide H_2_O_2_ were demonstrated to mediate induced eosinophil apoptosis as well as spontaneous eosinophil apoptosis [52,68,78–81]. In most tissues, the mitochondrial electron transport chain and especially the complexes I and III serve as the primary source of superoxide (O_2_^•−^) even thought O_2_^•−^ can also be generated by function of NADPH oxidase or xanthine oxidase in certain immune cells such as eosinophils, neutrophils and macrophages following their activation [82]. Approximately five- to ten-fold higher steady state concentrations of O_2_^•−^ exist in the mitochondrial matrix when compared to the cytosol, according to one estimation [83]. Mitochondria have a diverse antioxidant defence system including superoxide dismutases (SODs such as Manganese (Mn)-containing SOD), glutathione, glutathione peroxidase, catalase, peroxiredoxins *etc*. In the presence of superoxide dismutase (SOD), O_2_^•−^ is converted into a more stable non-radical oxidant, hydrogen peroxide (H_2_O_2_) that may also function as a signalling molecule [84]. Catalase functions by decomposing H_2_O_2_ to oxygen and water.

### 3.1. ROS

H_2_O_2_ has been shown to induce eosinophil apoptosis and catalase has been demonstrated to decrease spontaneous eosinophil apoptosis [55,85]. Eosinophil apoptosis induced by excretory-secretory products from helminth was associated with increased levels of H_2_O_2_ but not superoxide and reversed by catalase but not by mimetic of superoxide dismutase (SOD). Increased H_2_O_2_ preceded mitochondrial injury [86]. It is possible that H_2_O_2_ often acts as the actual mediator of eosinophil apoptosis instead of O_2_^•−^. H_2_O_2_-induced apoptosis also required products of the mitochondrial respiratory chain because inhibition of mitochondrial respiration by rotenone decreased H_2_O_2_-induced eosinophil apoptosis [55]. In thymocytes, glucocorticoids induced production of H_2_O_2_ and overexpression of catalase in these cells resulted in their resistance to glucocorticoid-induced apoptosis supporting a mediator role of H_2_O_2_ in the process [87,88]. In eosinophils, evidence exists also for the important role of superoxide itself as the mediator of apoptosis. Glucocorticoids increased levels of superoxide in eosinophils after 24 h of treatment [52]. Spontaneous apoptosis as well as glucocorticoid-induced apoptosis were associated with decreased level of the mitochondrial antioxidant MnSOD but not that of the cytosolic antioxidant CuZnSOD at 24 h [52]. Decreased level of MnSOD would lead to increased levels of superoxide radical and decreased formation of H_2_O_2_. Also, nitric oxide-induced eosinophil apoptosis was reduced by a SOD mimetic, suggesting that superoxide is an important mediator [68]. Why are the levels of ROS elevated when eosinophils are on their way towards apoptosis? Generally, it is thought that most often increased levels of ROS result from compromised antioxidant capacity rather than increased production of superoxide [82,89]. Indeed, as discussed above, glucocorticoids were shown to decrease levels of MnSOD in eosinophils [52]. The mechanism of glucocorticoid-induced enhancement of ROS has also been studied in many cell types other than eosinophils. In thymocytes, glucocorticoid-induced production of H_2_O_2_ was dependent on complex III of the mitochondrial respiratory chain and these events mediated glucocorticoid-induced apoptosis. In isolated mitochondria, glucocorticoids have also been shown to inhibit members of the mitochondrial respiratory chain [90,91]. In neural stem cells, treatment with dexamethasone resulted in down-regulation of 72% of the investigated genes involved in the mitochondrial respiratory chain, as well as 29% of the genes encoding for antioxidant enzymes [92]. Altogether, the source of ROS production may vary depending on the pro-apoptotic stimulant. Glucocorticoids may elevate ROS by directly modulating function of the enzymes in the mitochondrial electron transport chain as well as by transcriptional regulation of antioxidant or respiratory chain enzymes.

ROS are significant mediators of eosinophil apoptosis induced by many pro-apoptotic agents but how do they actually mediate eosinophil cell death? At some point, excessive mitochondrial ROS may reach a threshold that leads to mPT pore opening resulting in ROS release to the cytosol [76]. In the cytosol, ROS may activate several protein kinases as discussed below. According to the hypothesis of Zorov *et al.* the released ROS might trigger a similar phenomenon in neighbouring mitochondria and lead to amplified oxidative stress signals, mitochondrial injury and possibly cell death [76]. Alternatively, a mPT-independent mechanism was demonstrated where ROS was required for mitochondrial membrane permeabilization and cytochrome c release induced by Bid [70].

### 3.2. Kinases Activated by ROS

Mitogen-activated protein kinases (MAPKs) are serine/threonine kinases mainly activated by bacterial products, proinflammatory cytokines, growth factors and environmental stress. MAPK family consists of c-jun *N*-terminal kinase (JNK) 1–3, extracellular regulated kinase (ERK) 1/2, 3, 5 and 7, and p38 kinases. A phosphorylation cascade conducted by MAPK kinase kinases (MAP3K) and MAPK kinases (MAP2K) leads to activation of MAPK. MAPKs phosphorylate transcription factors resulting in transcription of genes involved in apoptosis, survival, proliferation and differentiation. Additionally, MAPKs affect the function of numerous other proteins via phosphorylation. MAPKs are inactivated by phosphoprotein phosphatases (MAPK phosphatases (MKPs)) [93–95]. Germinal center kinases (GSK) such as mammalian sterile 20-like kinase (Mst) 1 are also activators of MAPK pathways and at least some family members function by acting as MAPK kinase kinase kinases (MAP4K) [96].

#### 3.2.1. JNK

ROS are known activators of MAPKs JNK, p38 and ERK 1/2, known to regulate cell survival and death pathways [97]. Of these MAPKs, JNK has been shown to mediate glucocorticoid-induced eosinophil apoptosis as well as spontaneous apoptosis, apoptosis induced by nitric oxide and several drugs [27,52,68,98–101]. Activation of JNK by ROS seems to be indirect. Even though not shown in eosinophils, the actual targets of oxidants such as MAP3K (MEKK1) and apoptosis signal-regulating kinase 1 (ASK1 or MAP3K5) reside upstream of JNK, and lead to JNK activation via function of MAP2K [97]. In addition, peroxynitrite led to activation of JNK in eosinophils but was found to require Fas. Fas was demonstrated to be a direct oxidation target of reactive nitrogen species (RNS) but the mechanism of JNK activation via Fas remains unclear [102].

Kinetics of JNK activation seems to be an important determinant in whether the activation leads to survival or apoptosis. Early JNK activation has been described to represent a stress response resulting in cell survival signalling while delayed and sustained JNK activation has been typically related to apoptosis [97,103,104]. Indeed, early and strong JNK activation was demonstrated as a feature preceding glucocorticoid-induced eosinophil apoptosis, as well as apoptosis induced by several other factors in eosinophils [52,68,101]. Eosinophil apoptosis induced by glucocorticoids and nitric oxide exhibit many similarities such as early and late JNK activation, mPT and caspase activation [52,68]. Early JNK activation induced by nitric oxide, was however, not critical for NO-induced apoptosis and might initiate a stress response aiming to cell survival [68]. In the study of Gardai *et al.*, early JNK phosphorylation stimulated by glucocorticoids was prevented by antioxidant treatment suggesting involvement of ROS [52]. Furthermore, in NO-treated eosinophils, early JNK activation was dependent on partial mitochondrial permeability transition (mPT) [68]. By combining these results with findings of Zorov *et al*. it can be suggested that early JNK activation in response to glucocorticoids could be mediated by mPT stimulated by ROS and the following release of ROS to the cytosol [76]. Activation of JunD by JNK might provide a link to cell survival [105].

**Figure 1.**
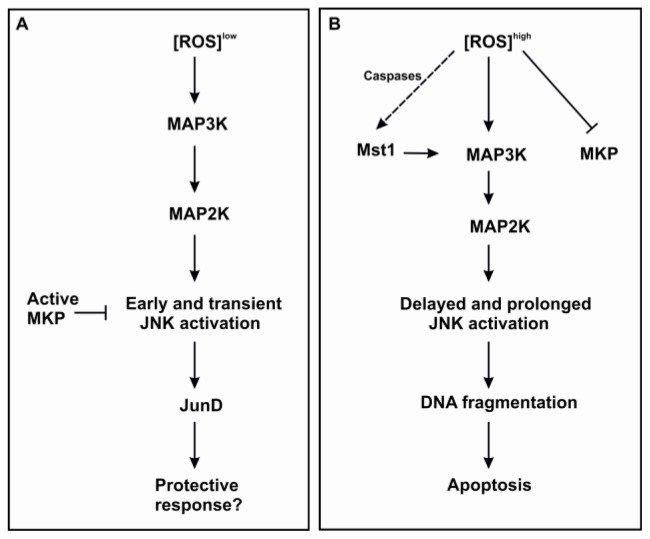
Hypothetical model to describe the mechanisms and outcomes of MAPK activation induced by different levels of ROS. If a pro-apoptotic stimulant induces release of ROS that is high enough to quench MKP, this may lead to prolonged JNK activation and apoptosis (**B**); Low levels of ROS may lead to transient activation of JNK due to presence of active MKP and end up in a protective response (**A**). The specific pathway mediating activation of Mst1 is unclear. ROS, reactive oxygen species; MAP3K, mitogen-activated protein kinase kinase kinase; MAP2K, mitogen-activated protein kinase kinase; JNK, c-Jun *N*-terminal kinase; MKP, MAP kinase phosphatase; Mst1, mammalian sterile 20-like kinase 1.

Instead, an additional later and sustained activation phase of JNK seems to take place in eosinophils undergoing glucocorticoid- and NO-induced apoptosis [52,68] and evidence exists that the late phase mediates apoptosis [68,103,104]. Interestingly, it was shown, that ROS oxidizes the inactivators of MAPKs, MAP kinase phosphatases (MKPs), thereby inhibiting their action and enabling prolonged JNK activation [106]. It is possible that only high levels of ROS lead to oxidation and inactivation of MKPs and prolonged JNK activation while low levels of ROS may not influence MKP activity resulting in rapid JNK inactivation (Figure 1). This could explain the kinetics of JNK activation in eosinophils. In eosinophils, JNK may be mainly involved in regulating DNA fragmentation, because its inhibition prevented DNA fragmentation but not e.g., phosphatidylserine exposure or morphological signs in induced apoptosis [99,101].

#### 3.2.2. ERK

There remains some controversy whether ERK has a role in mediating cytokine-afforded eosinophil survival [57,107,108]. However, evidence exists for its role in mediating eosinophil cell death even though it was not involved in eosinophil apoptosis induced by dexamethasone [109]. Activation of ERK1/2 (but not ERK5) preceded H_2_O_2_-induced caspase activation and eosinophil apoptosis [55]. Additionally, Siglec-8 induced ROS-dependent cell death in IL-5-treated eosinophils that was mediated by enhanced activation of ERK1/2 [81]. However, siglec-8-induced cell death tended to be more necrotic than apoptotic. Recently, paired immunoglobulin-like receptor A (PIR-A) was demonstrated to drive eosinophils into apoptosis in the absence of its suppressor PIR-B. The pro-apoptotic activity of PIR-A was found to involve Grb2 association and ERK1/2 phosphorylation [110]. Furthermore, anti-CD30 antibody induced eosinophil apoptosis that was partially prevented by inhibitors of MAP/extracellular signal-regulated kinase kinase (MEK) 1 and MEK1/2 that lie upstream of ERK1/2 [111].

#### 3.2.3. p38

In eosinophils, MAPK p38 has been mainly shown to mediate survival rather than apoptosis [108,112,113]. However, eosinophil apoptosis induced by anti-CD30 antibody was partially prevented by SB203580, an inhibitor of p38 [111], suggesting that similarly to JNK and ERK, p38 may also act as mediator of apoptosis in certain circumstances. Whether ROS were involved in the activation of p38, remains to be determined.

#### 3.2.4. Mst 1/2

Mammalian sterile 20-like kinase (Mst) 1 belonging to the group of germinal center kinases (GSKs), is involved in many functions of immune cells including apoptosis [96]. Release of 36 kDa fragment of Mst1 correlated with eosinophil apoptosis and was inhibited by catalase and inhibitor of caspases [114], suggesting that Mst1 activation was dependent on H_2_O_2_ and caspases. In embryonic stem cells, Mst1 was demonstrated to be involved in activation of JNK and chromatin condensation during apoptosis. This effect was dependent on upstream activators of JNK, MAP2K4 and MAP2K7, because when these kinases were suppressed, Mst1 was not able to mediate chromatin condensation [115].

## 4. Summary and Conclusions

**Figure 2.**
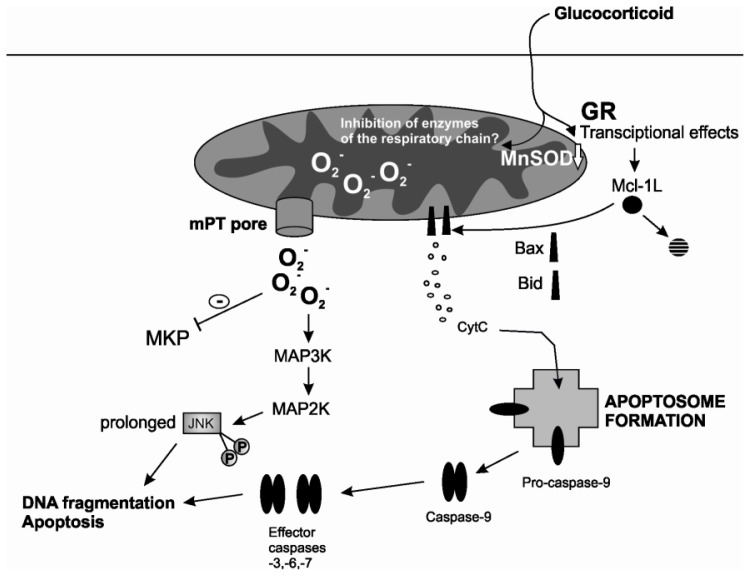
A proposed model of the pro-apoptotic mechanism of glucocorticoids in eosinophils. Glucocorticoid-induced eosinophil apoptosis is partially dependent on glucocorticoid receptor and stimulate an intrinsic pathway of apoptosis. Transcriptional effects may lead to reduced level of mitochondrial antioxidant MnSOD and elevated ROS. It is also possible that glucocorticoids directly inhibit mitochondrial respiratory chain enzymes. Elevated ROS may lead to mPT and release of ROS to the cytosol. ROS inhibits MKP and stimulates activation of the MAPK pathway. Glucocorticoids may also enhance degradation of anti-apoptotic Mcl-1L by a transcriptional route. Degradation of Mcl-1L enables oligomerization and pore-forming activity of Bax leading to mitochondrial outer membrane permeabilization. Processing of pro-apoptotic Bid into its truncated, pore-forming fragment is also enhanced by glucocorticoids. Abbreviations: GR, glucocorticoid receptor; Mcl-1L, myeloid cell leukemia 1 (long); MnSOD, manganese superoxide dismutase; mPT, mitochondrial permeability transition; CytC, cytochrome c; MKP, map kinase phosphatase; JNK, c-Jun *N*-terminal kinase; MAP3K, mitogen-activated protein kinase kinase kinase; MAP2K, mitogen-activated protein kinase kinase.

Mitochondria are extremely central in mediating induced eosinophil apoptosis and are involved in many steps from the early stress response to the decision of the cell to cope or undergo apoptosis as well as to the final loss of mitochondrial membrane potential. Many clinically relevant inducers of eosinophil apoptosis utilize the intrinsic pathway of apoptosis and even the extrinsic pathway stimulated by Fas activation involves a critical mitochondrial loop. Glucocorticoids and nitric oxide stimulate an intrinsic pathway with many similar features involving ROS, early and late JNK activation and mPT (Figure 2). Additionally, glucocorticoids accelerate degradation of anti-apoptotic Mcl-1 which might enable oligomerization and pore-forming activity of Bax. Processing of pro-apoptotic Bid into its truncated, pore-forming fragment is also enhanced by glucocorticoids. Studies support that ROS may have a central role in mediating many of these events: JNK activation, mPT induction and Bid-mediated cytochrome c release. Glucocorticoids decrease levels of mitochondrial antioxidants in eosinophils, which most likely enhance their pro-apoptotic effect. Understanding of these pathways in eosinophil apoptosis is critical to support development of new agents to treat eosinophilic disorders such as asthma. Furthermore, these pathways may also occur in other immune cells in response to glucocorticoids, and increase our understanding of the mechanisms behind the divergent effects of glucocorticoids on the longevity of different cell types.
